# Dietary Regulation of Histone Acetylases and Deacetylases for the Prevention of Metabolic Diseases

**DOI:** 10.3390/nu4121868

**Published:** 2012-11-28

**Authors:** Tho X. Pham, Jiyoung Lee

**Affiliations:** Department of Nutritional Sciences, University of Connecticut, 216 Advanced Technology Laboratory Building, 1392 Storrs Road, Storrs, CT 06269, USA; Email: Tho.Pham@uconn.edu

**Keywords:** high-fat, HDAC, HAT, sulforpahane, curcumin, butyrate, epigenetics

## Abstract

Age-related diseases such as type 2 diabetes, cardiovascular disease, and cancer involve epigenetic modifications, where accumulation of minute changes in the epigenome over time leads to disease manifestation. Epigenetic changes are influenced by life style and diets. This represents an avenue whereby dietary components could accelerate or prevent age-related diseases through their effects on epigenetic modifications. Histone acetylation is an epigenetic modification that is regulated through the opposing action of histone acetylases (HATs) and deacetylases (HDACs). These two families of enzymes play critical roles in metabolic processes and their dysregulation is associated with pathogenesis of several diseases. Dietary components, such as butyrate, sulforaphane, and curcumin, have been shown to affect HAT and HDAC activity, and their health benefits are attributed, at least in part, to epigenetic modifications. Given the decades that it takes to accumulate epigenetic changes, it is unlikely that pharmaceuticals could undo epigenetic changes without side effects. Therefore, long term consumption of dietary components that can alter the epigenome could be an attractive means of disease prevention. The goal of this review is to highlight the roles of diets and food components in epigenetic modifications through the regulation of HATs and HDACs for disease prevention.

## 1. Introduction

Obesity is characterized by an abundance of energy that exceeds the body’s needs. While a decline in physical activity is partially to blame, the consumption of diets rich in calories, particularly high in simple sugar and fat, appears to be a major contributor to the development of obesity [1]. The prevalence of obesity and its associated metabolic and cardiovascular dysfunction, termed “metabolic syndrome”, have led to increased morbidity and mortality [2,3,4]. The increased recognition that the development of metabolic syndrome involves epigenetic mechanisms has shed new light onto the intergenerational transmission of metabolic syndrome [5,6]. Although this has highlighted the importance of maternal diets and their effect on the transmission of metabolic syndrome, the underlying molecular mechanism for diet-induced epigenetic changes requires further investigation. Dietary components that are known to affect epigenetic modifying enzymes represent an invaluable tool to determine the effects of diets on epigenetic changes and whether the changes contribute to a favorable metabolic phenotype. 

Epigenetics refers to the studies of heritable changes in gene expression that occur without a change in underlying DNA sequence [7]. Central to epigenetics are DNA and histone modifications that alter gene transcription, but now also encompasses microRNA [7]. DNA methylation is perhaps the best understood epigenetic modification, whereby methylation at CpG dinucleotides mediate transcriptional silencing of methylated genes [8]. It is increasingly evident that DNA methyltransferases do not act alone, but also recruit histone deacetylases (HDACs) to synergistically repress gene transcription [9,10]. DNA methylation is a more stable modification compared to histone acetylation as evidenced by a lack of a direct DNA demethylating enzyme [11,12]. The relative irreversibility of methylation makes dietary regulation of DNA methylation less appealing. Therefore, the regulation of histone acetyltransferases (HATs) and HDACs represents an attractive means by which dietary components can have an impact on the epigenome. The goal of this review is to highlight the roles of diets and food components in epigenetic modifications through the regulation of HATs and HDACs for disease prevention.

## 2. Histone Acetyltransferases (HATs) and Histone Deacetylases (HDACs)

HATs and HDACs are enzymes that facilitate the addition or removal of acetyl moieties from lysine residues on histone tails, respectively. The addition of an acetyl moiety by HATs to the ε-amino group of lysine neutralizes its positive charge, decreasing the electrostatic interaction between the negatively charged DNA backbone and histone tails. This, in turn, relaxes chromatin structure and allows access of transcription factors to target gene promoters [13]. In general, hyperacetylation of histone tails induces transcriptional activation while hypoacetylation is associated with transcriptional repression [14]. 

In humans, HATs can be categorized into four families. Members within each family share significant protein sequence homology, while members between family have very little sequence similarities [15]. Members of the general control non-derepressible 5 (Gcn5)-related *N*-acetyltransferase (GNAT) family consist of Gcn5 and its ortholog p300/CREB-binding protein (CBP)-associated factor (PCAF) [16]. The MYST family, named for its founding members such as MOZ, Ybf2/Sas3, Sas2, and Tip60, includes monocytic leukemia zinc finger protein (MOZ), TAT-interactive protein with mass of 60 kDa (TIP60), MOZ-related factor (MORF), and HAT bound to ORC1 (Hbo1) [16]. The p300/CBP family consists of p300 and CBP. The fourth family of HAT is the steroid receptor coactivator (SRC) family that consists of SRC-1, nuclear receptor coactivator (ACTR), SRC-3, and TATA box-binding (TBP)-associated factor (TAF) [15,16,17]. 

Mammalian HDACs are categorized into 2 families and 4 classes. The classical zinc-dependent HDACs of the Rpd3/Hda1 family is made up of class I, II and IV, while the NAD^+^-dependent sirtuin family is categorized as class III HDACs [14]. Class I HDACs include HDAC1, 2, 3, and 8. Class II HDACs are further subcategorized into class IIa and IIb. Members of class IIa are HDAC4, 5, 7, and 9, while members of the class IIb include HDAC6 and 10. Class III HDACs are SIRT1 through 7 and class IV is HDAC11 [14,18]. 

Acetylation of histones by HATs is closely tied to acetyl-CoA availability as the acetyl group donor [19]. Distinct pools of acetyl-CoA exist for acetylation in cells: only the cytoplasmic and nuclear pools of acetyl-CoA are used for acetylation by HAT, while mitochondrial acetyl-CoA is used for energy production [19,20]. The deacetylation of histones by HDACs releases free acetate, which is used to regenerate acetyl-CoA after being ligated to free CoA by acetyl-CoA synthetase1 and 2 [21]. Interestingly, enzyme kinetic studies using recombinant HDAC1 and 2 showed that HDAC catalytic activity can be increased by intermediates such as acetyl-CoA, malonyl-CoA, and HMG-CoA, but inhibited by free CoA [22]. The studies suggest that intermediates of metabolism can directly regulate HDAC activity. 

In addition to the regulation of HAT and HDAC activity by substrate availability, subcellular localization is another important mechanism by which HAT and HDAC activities are regulated. It has been reported that PCAF and human Gcn5 are phosphorylated by calcium-dependent kinases before being translocated to the nucleus in PC12 cells and inhibition of calcium-dependent kinases led to accumulation of PCAF and Gcn5 in the cytoplasm [23]. This nuclear translocation of PCAF was also shown to depend on autoacetylation of the PCAF nuclear localization sequence because PCAF mutants lacking HAT activity were found to be in the cytoplasm [24]. The subcellular localization of HDACs has been more understood than HATs. Generally it is believed that Class I HDACs are predominately found in the nucleus, while Class II HDACs translocate in and out of the nucleus depending on cellular signaling [14]. A well-known exception is HDAC3. Although HDAC3 is a Class I HDAC, it possesses a nuclear export sequence, suggesting it can translocate to the cytoplasm [25]. Indeed, HDAC3 was found in the cytoplasmic fraction of 3T3-L1 adipocytes and was enriched in the nuclear fraction upon stimulation by tumor necrosis factor α (TNFα) [26]. To our knowledge, well-known examples of HDACs whose activity is primarily regulated via alterations in cellular translocation are HDAC4 and 5. Upon phosphorylation by calcium/calmodulin-dependent protein kinases, HDAC4 and 5 are bound by 14-3-3 protein and exit from the nucleus [27,28].

## 3. Diet-Induced Epigenetic Changes

### 3.1. High Fat Diet

The transition from a lean to an obese state is accompanied by marked changes in gene expression, at least in part, as an adaptive mechanism to excess calories [29]. Some of these changes in gene expression occur through epigenetic mechanisms induced by a high fat diet. Evidence from the studies on the effect of maternal nutrition on susceptibility of offspring to metabolic diseases supports the notion that consumption of a high fat diet during pregnancy is likely to induce epigenetic changes, predisposing offspring to metabolic syndrome later in life. Offspring of female rats consuming a high fat diet during pregnancy developed metabolic syndrome after birth even when they were weaned onto a chow diet [30] (For details of transgenerational studies see Table 1). Similarly, high fat-induced predisposition of offspring to metabolic syndrome was also found in mice [31,32]. Furthermore, insulin resistance was detected in the second generation of mice that were fed a normal chow diet [31]. In Japanese macaques that were fed a high fat diet (35% calories from fat) during pregnancy, fetal offspring had increased H3 acetylation and decreased HDAC1 expression in the liver compared to that of macaques fed a low fat diet (13% calories from fat) [33]. In humans, positive association was found between parental weight and incidence of obesity in offspring [34]. Although the molecular mechanism by which high fat diet predisposes offspring to metabolic syndrome remains elusive, the observations that early exposure to a high fat diet during fetal development affects disease susceptibility of offspring at a much later time, even after the removal of the initial stimulus, suggest epigenetic changes occurred during pregnancy in response to maternal diets. 

**Table 1:** Summary of transgenerational mice studies.

Diets		Model	Study Design	Route of Administration	Endpoint Measurements	Authors
*High Fat Diet*	Chow (12% fat, 65% carbohydrate, 23% protein) *vs.* High fat (59% fat, 20% carbohydrate, 21% protein)	Wistar rats	Male and female rats were kept on a chow or high fat diet for 4 weeks and bred. Respective diets were continued throughout pregnancy and stopped at lactation. At 21 days, all pups were weaned onto a chow diet. Male offsprings were studied at 3 months of age.	*Ad libitum*	Body composition, glucose tolerance, insulin sensitivity, respiratory quotient, insulin receptor expression	Buckley, A.J. *et al.* [30]
	Chow (12% fat, 28% protein, 60% carbohydrate) *vs.* High fat (34% CHO, 45% fat, 20% casein)	C57BL/6: 129 hybrid	Female mice on chow or high-fat diet were bred to produce F1 offsprings. F1 offsprings on each diet were then bred to create four combination of F2 mice: F2 never exposed to high-fat diet from both maternal or paternal sources (chow), F2 mice exposed to high fat diet from paternal sources, F2 mice exposed to high fat diet from maternal source and F2 mice exposed to high fat diet both from paternal and maternal sources.	*Ad libitum*	Body composition, glucose tolerance, insulin sensitivity, serum insulin levels	Dunn, G.A. *et al.* [31]
	Chow ( 13% fat) *vs.* High fat (35% fat)	Japanese macaques	Animals were naturally bred over four seasons. Pregnancies were terminated by Cesarean at gestational day 130.	Not available	Liver histone acetylation, HDAC activity, liver gene expression	Aagaard-Tillery, K.M. *et al.* [33]
*High Glycemic Diet*	Not available	ICR mice	Pregnant ICR female mice divided into two groups: control and intrauterine hyperglycemia group with gestational hyperglycemia (GDM). Mice in the GDM group were injected with streptozotocin at a concentration of 150 mg/kg body weight. F1 control and GDM mice were then crossed to create 4 different F2 progeny: male and female F1 control mice, GDM F1 male and F1 female control mice, GDM F1 female mice and F1 male control mice, and male and female GDM F1 mice.	Not available	Glucose tolerance test, insulin tolerance test, insulin secretion, pancreas gene expression, DNA methylation	Ding, G.L. *et al.* [35]

The liver is a center of energy metabolism and the detrimental effect of a high fat diet can usually be observed in the liver. The liver, being the primary site of detoxification within the body, is under constant toxic challenges, making tissue regeneration vital for the maintenance of its function. Indeed, mice on a high fat diet for 9 weeks showed impaired liver regeneration following partial hepatectomy when compared to mice fed a normal chow diet [36]. Similar findings were also observed in male Sprague-Dawley rats on a high fat diet for 4 weeks, and interestingly, the regenerative ability of the liver is inversely correlated with hepatic HDAC1 protein levels [37]. It was shown that HDAC1 cooperates with CCAAT enhancer-binding protein α (C/EBPα) to inhibit liver proliferation in older mice [38]. Furthermore, loss of HAT-mediated histone acetylation in mice impaired liver regeneration [39]. This suggests that a high fat diet could modulate HAT and HDAC activity to regulate liver regeneration, which can have a large impact on liver function particularly in the status of disease and metabolic dysfunction. 

HDAC3 is involved in hepatic gluconeogenesis, a process that is dysregulated in metabolic syndrome induced by high fat diet [40]. It has been shown that HDAC3 is recruited to the promoter of lipogenic genes, such as fatty acid synthase (FAS) and stearoyl-CoA desaturase, to silence their expression and thus reroute metabolic precursors for gluconeogenesis during fasting [40]. Consistent with this finding, the HAT, PCAF was shown to increase FAS mRNA levels in the liver in conjunction with decreased HDAC9 expression in mice [41,42]. In a fed state, the absence of HDAC3 from the promoter of lipogenic genes allows for their transcription and thus increased lipogenesis [40]. When DA.1U rats and E3 rats, which have a distinctive genetic background from each other, were fed a high fat diet for 12 weeks, it was discovered that DA.1U rats were much more resistant to high fat-induced metabolic syndrome. It was shown that while a high fat diet increased hepatic HDAC3 expression in both strains of rats, the magnitude of HDAC3 expression was much less in DA.1U rats compared to E3 rats, suggesting that HDAC3 is responsible for the high fat-induced metabolic syndrome [43]. This result is in agreement with the finding that *Hdac3* liver-specific knockout mice on a high fat diet for 4 weeks were more insulin sensitive and had lower blood glucose levels than wild-type mice [40]. Taken together, the studies suggest that HDAC3 is up-regulated in response to a high fat diet. The elevated expression of HDAC3 in the liver leads to a decrease in insulin sensitivity through an unknown mechanism, but also leads to a repression of lipogenesis, which consequently promotes the liver to be more gluconeogenic. Although increased hepatic glucose production by HDAC3 has been shown to contribute to hyperglycemia seen in metabolic syndrome, given the repressive role of HDAC3 in lipogenesis, further study is necessary to evaluate the effect of HDAC3 in the different metabolic state of the body. 

### 3.2. High Glycemic Diet

Consumption of diets high in simple sugars or foods with a high glycemic index is associated with increased risk of metabolic diseases [44]. Foods with high glycemic index are readily absorbed, resulting in a rapid rise in blood glucose levels. Similar to high fat diets, high blood glucose levels in the intrauterine environment predispose offspring to metabolic syndrome [35]. When pregnant mice were injected with a single dose of 150 mg/kg body weight of streptozotocin to induce hyperglycemia in the intrauterine environment, the F2 offspring of these mice had increased birth weight and impaired glucose tolerance starting at 3 weeks of age [35]. This study suggests that predisposition to metabolic syndrome was inherited to the F2 offspring through an epigenetic mechanism.

C57BL/6 mice were fed an isoenergetic diet containing 65% carbohydrates with two different carbohydrate compositions: one contained 100% amylopectin and the other consisted of 30% amylopectin and 70% amylose for carbohydrate source, representing a high and low glycemic index diet, respectively [45]. Although overall body weights of the two groups were not statistically significant until the 20th week, significant increases in body fat mass was observed at the 5th week. Mice on the high glycemic index diet also demonstrated a significant reduction in glucose clearance followed by a glucose challenge, but no statistical differences were observed for insulin tolerance [45]. A study using 129SvPas mice fed low or high glycemic index diet showed similar results to those observed with C57BL/6 mice but a high glycemic index diet induced hyperinsulinemia much earlier than did C57BL/6 strain [46]. These studies showed that a high glycemic index diet can induce metabolic syndrome in two different strains of mice, but their regulation of insulin secretion in response to blood glucose seems to differ between mouse strains. Exposure to high glucose is cytotoxic to pancreatic β-cells [47]. Perhaps the time difference in induction of hyperinsulinemia between the two strains of mice may be due to better glucose tolerance by the pancreatic β-cells of C57BL/6 mice than those of 12svPas. 

Pancreatic duodenal homeobox 1 (PDX1) regulates insulin transcription in response to plasma glucose levels [48,49]. It interacts with p300 to acetylate the promoter region of the gene encoding insulin, inducing the transcription of insulin [49]. At low plasma glucose levels, PDX1 associates with HDAC1 and 2 to repress insulin transcription [48]. This provides a mechanism by which high blood glucose levels after consumption of a high glycemic index meal could contribute to transient hyperinsulinemia. Furthermore, the activity of PDX1 is important for pancreas regeneration because impairment of PDX1 activity decreases the ability for pancreatic β cells to generate mass as a compensatory response to decreased insulin signaling [50]. Another transcription factor that is important for glucose sensing for β cells is the hypoxia inducible factor (HIF)-1α [51]. High levels of glucose have been shown to destabilize HIF-1α independent of oxygen levels [52]. The mechanism by which glucose can do this is currently not well understood, but it thought that the formation of methylglyoxal from an abundance of glucose can inhibit HIF-1α transcriptional activity by interfering with the interaction of HIF-1α with its co-activator, p300 [52]. 

Taken together, the aforementioned studies suggest that high fat or a high glycemic index diets could contribute to the development of metabolic syndrome by mechanisms involving epigenetic changes and transmission. The effects of a high fat diet on epigenetic changes are better understood than those of high glycemic index diets. Although these two diets are different in their composition, they gave rise to similar phenotypes in mice, suggesting they are likely to utilize a similar mechanism of action to induce metabolic syndrome. Perhaps the effects of these two diets are attributed to their ability to regulate HATs and HDACs. How high fat and high glycemic index diets regulate HATs and HDACs needs further investigation.

## 4. Bioactive Compounds That Regulate HATs and HDACs

### 4.1. Sulforaphane

Sulforaphane is an isothiocynate that can be found in cruciferous vegetables such as broccoli, cabbage, and kale [53]. Induction of the expression of the phase II detoxification enzyme nicotinamide adenine dinucleotide phosphate (NADPH): quinone oxireductase by sulforaphane was discovered in broccoli extract [54]. It has been shown that sulforaphane can also induce glutathione transferase expression in the murine liver cell line, Hepa1c1c7 [55]. The induction of phase II detoxification enzymes by sulforaphane is due to its ability to activate nuclear factor E2-related factor 2 (NRF2), which plays a central role in the regulation of basal and inducible expression of phase II detoxification enzymes as well as endogenous antioxidant mechanism [56]. NRF2 is normally localized in the cytoplasm where it is bound by Kelch-like ECH-associated protein 1 (Keap1). Keap1 limits the activity of NRF2 by sequestering it in the cytoplasm and promoting its proteasomal degradation. In response to oxidative stress, NRF2 dissociates from Keap1 and then translocate to the nucleus where it binds to antioxidant response elements (ARE) in the promoters of anti-oxidant enzymes [57]. NRF2 knockout mice showed impaired anti-oxidative responses and increased inflammation, resulting in increased intestinal carcinogenesis [58]. In athymic mice, oral administration of 12 mg/kg body weight of sulforaphane twice a day for 5 weeks after xenografts significantly reduced tumor size and increased apoptosis in the tumor [59]. Thus, studies have suggested that the anti-cancer properties of sulforaphane can be attributed to its ability to prevent oxidative stress-induced inflammation through the activation of NRF2 pathway [56,58,59]. 

Inhibitory effects of sulforaphane on HDAC activity may also contribute to its anti-cancer activities. It has been demonstrated that HCT116 colon cells and human embryonic kidney (HEK) 293 cells treated with 3–35 µM of sulforaphane showed a dose-dependent decrease in HDAC activity [60,61]. The inhibition of HDAC activity was suggested to be due to decreases in HDAC2 and 3 protein levels in HCT116 cells [61]. Furthermore, when mice were gavaged with a single dose of 10 µM sulforaphane, HDAC activity in the colonic mucosa was acutely inhibited within 6 h of treatment with concomitant increases in histone H3 and H4 acetylation [62]. Long-term supplementation of 443 mg/kg diet of sulforaphane for 10 weeks increased H3 and H4 acetylation in the ileum, colon, prostate, and peripheral blood mononuclear cells isolated from C57BL/6J mice [62]. Sulforaphane-*N*-acetylcysteine and sulforaphane-cysteine, metabolites of sulforaphane generated through the mercapturic acid pathway, have been suggested to mediate the inhibitory effect of sulforaphane on HDAC activity [60,62]. 

Recent studies suggest that HDAC inhibition plays a role in the activation of NRF2 [63,64]. In primary microglial cells isolated from Sprague-Dawley rats, treatment of 1 mM of valporic acid or 10 nM trichostatin A (TSA) for 24 h increased acetylated H3 and H4 with a concomitant increase in luciferase activity driven by an ARE promoter [63]. In RAW 264.7 macrophages, treatment of 30 nM of TSA for 16 h decreased the protein levels of Keap1, allowing NRF2 to translocate to the nucleus, and NRF2 binding to the promoter of heme oxygenase 1 was also increased [64]. As sulforaphane is known to inhibit HDAC activity and also to induce NRF2 activity, it is likely that sulforaphane works similarly as HDAC inhibitors such as valporic acid and TSA. Further study is necessary to address this possibility. 

Studies have shown that sulforphane also inhibits adipocyte differentiation. Treatment of 20 µM of sulforphane in 3T3-L1 preadipocytes inhibited adipocyte differentiation by inhibiting C/EBPβ early in the differentiation program and thus blocked mitotic clonal expansion [65]. The development of adipocytes is important for proper storage of extra triglycerides in adipose tissue to prevent ectopic fat disposition in other tissues. Failure to store triglycerides in adipose tissue is linked to the development of systemic insulin resistance and furthermore type 2 diabetes [66]. During adipocyte differentiation, HDAC1, 2 and 5 are down-regulated, leading to hyperacetylation in the promoter regions of adipogenic genes such as FAS, fatty acid binding protein 4, and peroxisome proliferator-activated receptor γ (PPARγ) [67]. Specifically, HDAC1 represses transcriptional activity of PPARγ, a master regulator of adipocyte differentiation and maturation in 3T3-L1 cells [67]. It has also been shown that HDAC9 is down-regulated during adipocyte differentiation with a concomitant increase in p300 at the promoter of C/EBPα, another transcription factor that plays a critical role in adipocyte differentiation [68]. Although it appears that HDACs negatively regulate adipocyte differentiation, inhibition of HDACs by several inhibitors did not increase adipocyte differentiation, but rather inhibited it [69,70]. Reasons for these puzzling observations have not been understood. The finding that sulforaphane inhibits adipocyte differentiation could be viewed negatively as enhanced adipocyte differentiation can prevent ectopic deposition of extra triglyceride into other tissues by storing more triglycerides in adipose tissue. This would pose as a challenge for the use of sulforaphane in preventative measures. It is worthy to note that to our knowledge no studies have shown that sulforaphane would inhibit adipogenesis *in vivo*. The *in vivo* effects of sulforaphane on adipogenesis have been very limitedly studied, but warrant further investigation. 

### 4.2. Curcumin

Curcumin is a polyphenol found in the popular spice turmeric. Biological functions of curcumin has been extensively studied and its anti-oxidant, anti-inflammatory, and anti-cancer properties are well known [71]. Although there are over 5000 publications that address certain aspects of curcumin thus far, it was only discovered that curcumin is a HAT inhibitor in 2004 [72]. In particular, curcumin is a specific inhibitor of p300/CBP but not PCAF [72]. Furthermore, the inhibitory role of curcumin in regulating p300/CBP was attributed to its ability to induce apoptosis in cancer cells through the induction of caspase or p53 signaling [72,73]. 

In diabetic conditions, elevated blood glucose levels can activate inflammatory signaling in monocyte through nuclear factor kappa B (NF-κB) pathway [74]. NF-κB, a major transcription factor in mediating inflammatory responses, can associate with p300/CBP to induce inflammatory gene expression [75]. Curcumin inhibited p300/CBP activity and consequently decreased NF-κB transcriptional activity in human mantle cell lymphoma [76]. Furthermore, THP-1 monocytes exposed to hyperglycemic conditions, *i.e.*, 25 mM glucose, had decreased HDAC activity, but increased HAT activity when compared to monocyte cultured in normoglycemic conditions (5.5 mM glucose) [77]. The high glucose-induced alterations in HAT and HDAC activity increased NF-κB activity and the production of interluekin-6 and TNFα, which was abrogated by curcumin [77]. Male Sparague Dawley rats fed a high fat diet for 60 days developed hyperglycemia, hyperinsulinemia and insulin resistance, but supplementation of 80 mg/kg/day of curcumin to the high fat diet maintained fasting glucose levels similar to those of rats fed a normal chow diet [78]. Curcumin supplementation was also able to increase insulin sensitivity while plasma concentrations of free fatty acids and TNFα were decreased [78]. The protection from high fat-induced insulin resistance by curcumin was attributed, at least in part, to its anti-inflammatory effect. Indeed, in streptozotocin-induced diabetic rats, intraperitoneal injection of 150 mg/kg/day of curcumin prevented elevation of endothelial nitric oxide synthase and transforming growth factor-β1 in the kidney by the inhibition of p300/CBP and NF-κB activity [79]. In prediabetic humans, none in the curcumin-treated group was diagnosed with diabetes after 9 month of the intervention, while 16.4% of the placebo group progressed from prediabetes to diabetes [80]. The curcumin-treated group had lower C-peptide levels, higher homeostasis model assessment (HOMA)-β, lower level of HOMA-IR, and higher plasma adiponectin levels, which suggests a better pancreatic β-cell function than the control group [80]. 

Curcumin appears to be beneficial for the prevention of high-fat induced metabolic syndrome. The mechanism by which curcumin conveys this protection is unclear, but its anti-inflammatory effects through the inhibition of p300 may play an important role in this protection. A major problem with the use of curcumin for the prevention of metabolic syndrome is its bioavailability. Curcumin is poorly bioavailable due to its low absorption in the intestine [71]. Enhancement of curcumin delivery by using capsules appears to circumvent its poor bioavailability and potentiates health benefits of curcumin in humans [80].

### 4.3. Epigallocatechin-3-gallate

Green tea is an ancient beverage derived from the plant *Camellia sinensis*. It is widely consumed around the world with many purported health benefits. Green tea is rich in flavonoid polyphenols of which the catechin, epigallocatechin-3-gallate (EGCG), has been attributed for many of green tea’s biological effects. EGCG represents 50%–75% of the total amount of catechin content, of which is 30%–40% of the dry weight of a cup of green tea [81,82]. 

Recently green tea polyphenols (GTP) have been shown to reduce HDAC activity in human prostate cancer LNCaP and PC-3 cells that have been treated with 10–80 µg/mL of GTP for 24 h. The ability of GTP to reduce HDAC activity in LNCap and PC-3 cells could be attributed to its ability to reduce class I HDAC protein levels, perhaps through proteasomal degradation [83]. Interestingly, screenings for HAT inhibitors have indicated EGCG as having potent anti-HAT activity, while other derivatives such as catechin, epicatechin, and epigallocatechin possess low HAT inhibitory activities [84]. EGCG was also shown to not possess any anti-HDAC activity [84]. It is likely that EGCG is more of a HAT inhibitor than it is an HDAC inhibitor since reduction in HDAC activity from GTP is a biological response leading to a reduction in HDAC protein levels. Subsequent loading of nuclear extracts for HDAC activity assays from GTP-treated LNCap and PC-3 cells would reflect the reduction in HDAC protein levels and result in what appears to be decreased HDAC activity [83]. The assay used to identify EGCG as a HAT inhibitor entails incubation of HeLa cell nuclear lysate with different amount of EGCG. Inhibition of HAT activity reflects interaction of EGCG with HAT enzymes, rather than a reduction of HAT enzymes [84]. Another explanation for the discrepancy between the effects of GTP and EGCG on HAT and HDAC is that GTP possesses other polyphenols which could be responsible for HDAC inhibition which EGCG cannot accomplish alone. 

Green tea polyphenols or EGCG have been shown to be beneficial in the prevention of metabolic syndrome in mice and rats. Seventeen weeks of supplementation of EGCG in male C57BL/6 mice on a high-fat Western diet with or without 3.2 g EGCG/kg body weight resulted in reduced body weight gain, lower fasting blood glucose, and a lower inflammatory profile with lower levels of serum cytokine such as IL-6 and granulocyte colony-stimulating factor [85]. The reduction in obesity-associated inflammation could be due to the inhibitory effects of EGCG on HAT activity. It was demonstrated that inhibition of HAT activity by EGCG led to decreased NF-κB acetylation. This led to a decrease in NF-κB activity with concomitant decreases in p300 at the promoter of IL-6 [84]. There is evidence to show that NF-κB is activated in an acetylation-dependent manner, therefore a reduction in NF-κB acetylation would result in a decreased inflammatory response [86]. The protective effects of EGCG against high fat-induced metabolic syndrome was also found in female Sprague Dawley rats fed a high fat diet, but was supplemented without or without 0.5% (wt/vol) GTP in their drinking water for 8 weeks. For every 1000 mg of supplemented GTP, there was 464 mg of EGCG [87]. After 8 weeks, rats on a high fat diet supplemented with GTP were found to have a significant reduction in body weight compared to rats that were not supplemented with GTP. Measurement of cytokines such as IL-1β and IL-6 from serum found significant decreases of both cytokines from rats supplemented with GTP [87].

Green tea polyphenols, in particular, EGCG appears to be an inhibitor of HAT activity. This provides a molecular mechanism in which EGCG can directly attenuate chronic inflammation, which is an underlying cause of metabolic syndrome [66]. It is apparent from the aforementioned studies that EGCG supplementation has protective effects against high fat-induced metabolic syndrome. One hindrance in the use of EGCG supplementation for the prevention of metabolic diseases is reports of hepatotoxicity which could be due to the pro-oxidant effects of EGCG at high doses [88]. While it appears to happen, hepatotoxicity has only been reported in response to use of green tea supplementation as a weight loss product in humans and is still relatively rare [89].

### 4.4. Butyrate

The gut microbiota is responsible for the formation of short chain fatty acid (SCFA) through the fermentation of dietary fibers. In general, SCFA includes acetate, propionate, and butyrate in the ratio of 60:25:15 in humans [90]. As SCFA production is limited to the gut in humans, research has primarily focused on the role of SCFA in gastrointestinal diseases. However, SCFA derived from the gut is also found in the systemic circulation [91]. Although the major source of butyrate is from the fermentation of dietary fibers, butyrate can also be found in butter and cheese [92]. The ability for butyrate to inhibit HDACs was initially discovered in 1978 [93]. While inhibition of HDACs is a common mechanism to inhibit cancer growth, the effect of butyrate on the prevention of colorectal cancer has been inconsistent [94]. The discrepancy in findings can be attributed to differences in the energy status of cells used in studies because butyrate can be used as an energy source by nutrient-deficient cells to proliferate, but it inhibits cell proliferation when there is sufficient energy [94].

When C57BL/6J mice were fed a high fat diet supplemented with 5% butyrate by weight, they had higher energy expenditures than control mice evidenced by lower body weight despite higher caloric intake in butyrate-fed mice than control mice [92]. Furthermore, there were increased PPARγ coactivator 1α (PGC-1α) and uncoupling protein 1 (UCP-1) mRNA and protein levels in brown adipose tissues of butyrate-fed mice, suggesting that butyrate may increase thermogenesis to dissipate energy as a heat. Butyrate-fed mice also showed better oral glucose tolerance and lower fasting glucose and insulin levels. The effects of 5% butyrate (wt/wt) supplementation on the prevention of glucose intolerance and insulin insensitivity induced by high fat feeding was also observed in C57BL/6N mice [95]. In another study, C57BL/6J mice on a high fat diet for 10 weeks were given 2 g/kg of teributyrin, a butyrate prodrug, 3 times a week [96]. Teributyrin supplementation decreased body weight gain while attenuating insulin resistance with decreased fasting plasma glucose and insulin levels. Interestingly, high fat diet-induced obese C57BL/6J mice injected with 500 mg/kg body weight of sodium butyrate inhibited HDAC3 activity in the liver [97]. The inhibition of HDAC3 activity induced the expression of fibroblast growth factor 21 (FGF21), resulting in increased fatty acid oxidation and ketone production [97]. FGF21 has been shown to increase PGC-1α expression, fatty acid oxidation, and TCA cycle flux in the liver [98]. Furthermore, it has been reported that butyrate can also induce NRF2 in IEC-6 cells, a small intestine epithelial cell line, and HT-29 cells, a human colonic adenocarcinoma cell line [99]. The molecular mechanism by which butyrate induces NRF2 activity could be similar to sulforaphane, which is known to inhibit HDAC activity. 

Taken together, these studies suggest that butyrate can be protective of high fat-induced glucose intolerance and insulin insensitivity. The molecular mechanism by which butyrate confers these protective properties remain to be elucidated, but appears to involve the inhibition of HDAC3 activity in the liver and also the induction of PGC-1α expression in the liver and brown adipose tissue. The activation of NRF2 may also be responsible for the beneficial effect of butyrate, but needs further investigation. 

## 5. Conclusions

With advance in our understanding of the contribution of epigenetic modifications to the development of metabolic syndrome and insulin resistance, the roles of dietary components with HAT and HDAC regulating properties, such as curcumin, sulforaphane, and butyrate, in the pathogenesis of the metabolic diseases have been recognized. Studies have suggested that supplementation of these components can help ameliorate high fat-induced inflammation, obesity, glucose intolerance, and insulin insensitivity. Although the molecular mechanisms underlying the inhibitory roles of curcumin, sulforaphane, and butyrate in the development of high fat-induced metabolic syndrome are unclear, the effects are, at least in part, through modifications in the expression and activity of HATs and HDACs.

While this review has focused on the potential role of these dietary components to alter the epigenome, it cannot be ruled out that they may confer their effects through acetylation and deacetylation of metabolic enzymes in addition to their roles in histone proteins. Accumulating evidence indicates that many enzymes involved in glucose and lipid metabolism are acetylated, which affects their activities [100,101]. Both HATs and HDACs are known to act on non-histone proteins, indicating that inhibition of HAT or HDAC activity may not only affect histone acetylation, but also metabolic enzymes [102]. It is currently unclear whether curcumin, sulforaphane or butyrate mediates their effect by altering the acetylation state of metabolic enzymes. This represents an area that needs further investigation, which would shed new light on the mechanism of action of these dietary components in the prevention of obesity-associated metabolic diseases.

## References

[1] Hill J.O., Wyatt H.R., Reed G.W., Peters J.C. (2003). Obesity and the environment: Where do we go from here?. Science.

[2] Ardern C.I., Janssen I. (2007). Metabolic syndrome and its association with morbidity and mortality. Appl. Physiol. Nutr. Metab..

[3] Isomaa B., Almgren P., Tuomi T., Forsen B., Lahti K., Nissen M., Taskinen M.R., Groop L. (2001). Cardiovascular morbidity and mortality associated with the metabolic syndrome. Diabetes Care.

[4] Ervin R.B. (2009). Prevalence of Metabolic Syndrome among Adults 20 Years of Age and over, by Sex, Age, Race and Ethnicity, and Body Mass Index: United States, 2003–2006; National Health Statistic Report No. 13.

[5] Slomko H., Heo H.J., Einstein F.H. (2012). Minireview: Epigenetics of obesity and diabetes in humans. Endocrinology.

[6] Poston L. (2011). Intergenerational transmission of insulin resistance and type 2 diabetes. Prog. Biophys. Mol. Biol..

[7] Goldberg A.D., Allis C.D., Bernstein E. (2007). Epigenetics: A landscape takes shape. Cell.

[8] Ng H.H., Bird A. (1999). DNA methylation and chromatin modification. Curr. Opin. Genet. Dev..

[9] Yang X., Phillips D.L., Ferguson A.T., Nelson W.G., Herman J.G., Davidson N.E. (2001). Synergistic activation of functional estrogen receptor (ER)-alpha by DNA methyltransferase and histone deacetylase inhibition in human ER-alpha-negative breast cancer cells. Cancer Res..

[10] Cameron E.E., Bachman K.E., Myohanen S., Herman J.G., Baylin S.B. (1999). Synergy of demethylation and histone deacetylase inhibition in the re-expression of genes silenced in cancer. Nat. Genet..

[11] Fritz E.L., Papavasiliou F.N. (2010). Cytidine deaminases: Aiding DNA demethylation?. Genes Dev..

[12] Inoue A., Shen L., Dai Q., He C., Zhang Y. (2011). Generation and replication-dependent dilution of 5fC and 5caC during mouse preimplantation development. Cell Res..

[13] Jenuwein T., Allis C.D. (2001). Translating the histone code. Science.

[14] De Ruijter A.J., van Gennip A.H., Caron H.N., Kemp S., van Kuilenburg A.B. (2003). Histone deacetylases (HDACs): Characterization of the classical hdac family. Biochem. J..

[15] Marmorstein R. (2001). Structure of histone acetyltransferases. J. Mol. Biol..

[16] Roth S.Y., Denu J.M., Allis C.D. (2001). Histone acetyltransferases. Annu. Rev. Biochem..

[17] Chen H., Lin R.J., Schiltz R.L., Chakravarti D., Nash A., Nagy L., Privalsky M.L., Nakatani Y., Evans R.M. (1997). Nuclear receptor coactivator ACTR is a novel histone acetyltransferase and forms a multimeric activation complex with P/CAF and CBP/p300. Cell.

[18] Gregoretti I.V., Lee Y.M., Goodson H.V. (2004). Molecular evolution of the histone deacetylase family: Functional implications of phylogenetic analysis. J. Mol. Biol..

[19] Wellen K.E., Thompson C.B. (2012). A two-way street: Reciprocal regulation of metabolism and signalling. Nat. Rev. Mol. Cell Biol..

[20] Wellen K.E., Hatzivassiliou G., Sachdeva U.M., Bui T.V., Cross J.R., Thompson C.B. (2009). ATP-citrate lyase links cellular metabolism to histone acetylation. Science.

[21] Sakakibara I., Fujino T., Ishii M., Tanaka T., Shimosawa T., Miura S., Zhang W., Tokutake Y., Yamamoto J., Awano M. (2009). Fasting-induced hypothermia and reduced energy production in mice lacking acetyl-CoA synthetase 2. Cell Metab..

[22] Vogelauer M., Krall A.S., McBrian M.A., Li J.Y., Kurdistani S.K. (2012). Stimulation of histone deacetylase activity by metabolites of intermediary metabolism. J. Biol. Chem..

[23] Wong K., Zhang J., Awasthi S., Sharma A., Rogers L., Matlock E.F., van Lint C., Karpova T., McNally J., Harrod R. (2004). Nerve growth factor receptor signaling induces histone acetyltransferase domain-dependent nuclear translocation of p300/CREB-binding protein-associated factor and hGCN5 acetyltransferases. J. Biol. Chem..

[24] Blanco-Garcia N., Asensio-Juan E., de la Cruz X., Martinez-Balbas M.A. (2009). Autoacetylation regulates P/CAF nuclear localization. J. Biol. Chem..

[25] Yang W.M., Tsai S.C., Wen Y.D., Fejer G., Seto E. (2002). Functional domains of histone deacetylase-3. J. Biol. Chem..

[26] Gao Z., He Q., Peng B., Chiao P.J., Ye J. (2006). Regulation of nuclear translocation of HDAC3 by ikappabalpha is required for tumor necrosis factor inhibition of peroxisome proliferator-activated receptor gamma function. J. Biol. Chem..

[27] McKinsey T.A., Zhang C.L., Lu J., Olson E.N. (2000). Signal-dependent nuclear export of a histone deacetylase regulates muscle differentiation. Nature.

[28] Grozinger C.M., Schreiber S.L. (2000). Regulation of histone deacetylase 4 and 5 and transcriptional activity by 14-3-3-dependent cellular localization. Proc. Natl. Acad. Sci. USA.

[29] De Fourmestraux V., Neubauer H., Poussin C., Farmer P., Falquet L., Burcelin R., Delorenzi M., Thorens B. (2004). Transcript profiling suggests that differential metabolic adaptation of mice to a high fat diet is associated with changes in liver to muscle lipid fluxes. J. Biol. Chem..

[30] Buckley A.J., Keseru B., Briody J., Thompson M., Ozanne S.E., Thompson C.H. (2005). Altered body composition and metabolism in the male offspring of high fat-fed rats. Metabolism.

[31] Dunn G.A., Bale T.L. (2009). Maternal high-fat diet promotes body length increases and insulin insensitivity in second-generation mice. Endocrinology.

[32] Masuyama H., Hiramatsu Y. (2012). Effects of a high-fat diet exposure *in utero* on the metabolic syndrome-like phenomenon in mouse offspring through epigenetic changes in adipocytokine gene expression. Endocrinology.

[33] Aagaard-Tillery K.M., Grove K., Bishop J., Ke X., Fu Q., McKnight R., Lane R.H. (2008). Developmental origins of disease and determinants of chromatin structure: Maternal diet modifies the primate fetal epigenome. J. Mol. Endocrinol..

[34] Lillycrop K.A. (2011). Effect of maternal diet on the epigenome: Implications for human metabolic disease. Proc. Nutr. Soc..

[35] Ding G.L., Wang F.F., Shu J., Tian S., Jiang Y., Zhang D., Wang N., Luo Q., Zhang Y., Jin F. (2012). Transgenerational glucose intolerance with igf2/H19 epigenetic alterations in mouse islet induced by intrauterine hyperglycemia. Diabetes.

[36] DeAngelis R.A., Markiewski M.M., Taub R., Lambris J.D. (2005). A high-fat diet impairs liver regeneration in C57BL/6 mice through overexpression of the NF-kappaB inhibitor, ikappabalpha. Hepatology.

[37] Tanoue S., Uto H., Kumamoto R., Arima S., Hashimoto S., Nasu Y., Takami Y., Moriuchi A., Sakiyama T., Oketani M. (2011). Liver regeneration after partial hepatectomy in rat is more impaired in a steatotic liver induced by dietary fructose compared to dietary fat. Biochem. Biophys. Res. Commun..

[38] Wang G.L., Salisbury E., Shi X., Timchenko L., Medrano E.E., Timchenko N.A. (2008). HDAC1 cooperates with c/ebpalpha in the inhibition of liver proliferation in old mice. J. Biol. Chem..

[39] Shukla V., Cuenin C., Dubey N., Herceg Z. (2011). Loss of histone acetyltransferase cofactor transformation/transcription domain-associated protein impairs liver regeneration after toxic injury. Hepatology.

[40] Sun Z., Miller R.A., Patel R.T., Chen J., Dhir R., Wang H., Zhang D., Graham M.J., Unterman T.G., Shulman G.I. (2012). Hepatic HDAC3 promotes gluconeogenesis by repressing lipid synthesis and sequestration. Nat. Med..

[41] Wong R.H., Sul H.S. (2009). DNA-PK: Relaying the insulin signal to USF in lipogenesis. Cell Cycle.

[42] Wong R.H., Chang I., Hudak C.S., Hyun S., Kwan H.Y., Sul H.S. (2009). A role of DNA-PK for the metabolic gene regulation in response to insulin. Cell.

[43] Li D., Wang X., Ren W., Ren J., Lan X., Wang F., Li H., Zhang F., Han Y., Song T. (2011). High expression of liver histone deacetylase 3 contributes to high-fat-diet-induced metabolic syndrome by suppressing the PPAR-gamma and LXR-alpha-pathways in E3 rats. Mol. Cell. Endocrinol..

[44] Brand-Miller J., Buyken A.E. (2012). The glycemic index issue. Curr. Opin. Lipidol..

[45] Isken F., Klaus S., Petzke K.J., Loddenkemper C., Pfeiffer A.F., Weickert M.O.  (2010). Impairment of fat oxidation under high- *vs.* Low-glycemic index diet occurs before the development of an obese phenotype. Am. J. Physiol. Endocrinol. Metab..

[46] Scribner K.B., Pawlak D.B., Aubin C.M., Majzoub J.A., Ludwig D.S. (2008). Long-term effects of dietary glycemic index on adiposity, energy metabolism, and physical activity in mice. Am. J. Physiol. Endocrinol. Metab..

[47] Donath M.Y., Ehses J.A., Maedler K., Schumann D.M., Ellingsgaard H., Eppler E., Reinecke M. (2005). Mechanisms of beta-cell death in type 2 diabetes. Diabetes.

[48] Mosley A.L., Ozcan S. (2004). The pancreatic duodenal homeobox-1 protein (Pdx-1) interacts with histone deacetylases Hdac-1 and Hdac-2 on low levels of glucose. J. Biol. Chem..

[49] Mosley A.L., Ozcan S. (2003). Glucose regulates insulin gene transcription by hyperacetylation of histone h4. J. Biol. Chem..

[50] Frogne T., Sylvestersen K.B., Kubicek S., Nielsen M.L., Hecksher-Sorensen J. (2012). Pdx1 is post-translationally modified *in vivo* and serine 61 is the principal site of phosphorylation. PLoS One.

[51] Cantley J., Grey S.T., Maxwell P.H., Withers D.J. (2010). The hypoxia response pathway and beta-cell function. Diabetes Obes. Metab..

[52] Bento C.F., Pereira P. (2011). Regulation of hypoxia-inducible factor 1 and the loss of the cellular response to hypoxia in diabetes. Diabetologia.

[53] Sasaki K., Neyazaki M., Shindo K., Ogawa T., Momose M. (2012). Quantitative profiling of glucosinolates by LC-MS analysis reveals several cultivars of cabbage and kale as promising sources of sulforaphane. J. Chromatogr. B Analyt. Technol. Biomed. Life Sci..

[54] Zhang Y., Talalay P., Cho C.G., Posner G.H. (1992). A major inducer of anticarcinogenic protective enzymes from broccoli: Isolation and elucidation of structure. Proc. Natl. Acad. Sci. USA.

[55] Posner G.H., Cho C.G., Green J.V., Zhang Y., Talalay P. (1994). Design and synthesis of bifunctional isothiocyanate analogs of sulforaphane: Correlation between structure and potency as inducers of anticarcinogenic detoxication enzymes. J. Med. Chem..

[56] McMahon M., Itoh K., Yamamoto M., Chanas S.A., Henderson C.J., McLellan L.I., Wolf C.R., Cavin C., Hayes J.D. (2001). The cap ‘n’ collar basic leucine zipper transcription factor Nrf2 (NF-E2 p45-related factor 2) controls both constitutive and inducible expression of intestinal detoxification and glutathione biosynthetic enzymes. Cancer Res..

[57] Copple I.M. (2012). The Keap1-Nrf2 cell defense pathway—A promising therapeutic target?. Adv. Pharmacol..

[58] Cheung K.L., Lee J.H., Khor T.O., Wu T.Y., Li G.X., Chan J., Yang C.S., Kong A.N. (2012). Nrf2 knockout enhances intestinal tumorigenesis in Apc(min/+) mice due to attenuation of anti-oxidative stress pathway while potentiates inflammation. Mol. Carcinog..

[59] Wang F., Shan Y. (2012). Sulforaphane retards the growth of UM-UC-3 xenographs, induces apoptosis, and reduces survivin in athymic mice. Nutr. Res..

[60] Myzak M.C., Karplus P.A., Chung F.L., Dashwood R.H. (2004). A novel mechanism of chemoprotection by sulforaphane: Inhibition of histone deacetylase. Cancer Res..

[61] Rajendran P., Delage B., Dashwood W.M., Yu T.W., Wuth B., Williams D.E., Ho E., Dashwood R.H. (2011). Histone deacetylase turnover and recovery in sulforaphane-treated colon cancer cells: Competing actions of 14-3-3 and Pin1 in HDAC3/SMRT corepressor complex dissociation/reassembly. Mol. Cancer.

[62] Myzak M.C., Dashwood W.M., Orner G.A., Ho E., Dashwood R.H. (2006). Sulforaphane inhibits histone deacetylase *in vivo* and suppresses tumorigenesis in Apc-minus mice. FASEB J..

[63] Correa F., Mallard C., Nilsson M., Sandberg M. (2011). Activated microglia decrease histone acetylation and Nrf2-inducible anti-oxidant defence in astrocytes: Restoring effects of inhibitors of HDACs, p38 MAPK and GSK3beta. Neurobiol. Dis..

[64] Wang B., Zhu X., Kim Y., Li J., Huang S., Saleem S., Li R.C., Xu Y., Dore S., Cao W. (2012). Histone deacetylase inhibition activates transcription factor Nrf2 and protects against cerebral ischemic damage. Free Radic. Biol. Med..

[65] Choi K.M., Lee Y.S., Sin D.M., Lee S., Lee M.K., Lee Y.M., Hong J.T., Yun Y.P., Yoo H.S. (2012). Sulforaphane inhibits mitotic clonal expansion during adipogenesis through cell cycle arrest. Obesity (Silver Spring).

[66] Guilherme A., Virbasius J.V., Puri V., Czech M.P. (2008). Adipocyte dysfunctions linking obesity to insulin resistance and type 2 diabetes. Nat. Rev. Mol. Cell Biol..

[67] Yoo E.J., Chung J.J., Choe S.S., Kim K.H., Kim J.B. (2006). Down-regulation of histone deacetylases stimulates adipocyte differentiation. J. Biol. Chem..

[68] Chatterjee T.K., Idelman G., Blanco V., Blomkalns A.L., Piegore M.G., Weintraub D.S., Kumar S., Rajsheker S., Manka D., Rudich S.M. (2011). Histone deacetylase 9 is a negative regulator of adipogenic differentiation. J. Biol. Chem..

[69] Catalioto R.M., Maggi C.A., Giuliani S. (2009). Chemically distinct hdac inhibitors prevent adipose conversion of subcutaneous human white preadipocytes at an early stage of the differentiation program. Exp. Cell Res..

[70] Lagace D.C., Nachtigal M.W. (2004). Inhibition of histone deacetylase activity by valproic acid blocks adipogenesis. J. Biol. Chem..

[71] Sharma R.A., Gescher A.J., Steward W.P. (2005). Curcumin: The story so far. Eur. J. Cancer.

[72] Balasubramanyam K., Varier R.A., Altaf M., Swaminathan V., Siddappa N.B., Ranga U., Kundu T.K. (2004). Curcumin, a novel p300/CREB-binding protein-specific inhibitor of acetyltransferase, represses the acetylation of histone/nonhistone proteins and histone acetyltransferase-dependent chromatin transcription. J. Biol. Chem..

[73] Kang S.K., Cha S.H., Jeon H.G. (2006). Curcumin-induced histone hypoacetylation enhances caspase-3-dependent glioma cell death and neurogenesis of neural progenitor cells. Stem. Cells Dev..

[74] Shanmugam N., Reddy M.A., Guha M., Natarajan R. (2003). High glucose-induced expression of proinflammatory cytokine and chemokine genes in monocytic cells. Diabetes.

[75] Gerritsen M.E., Williams A.J., Neish A.S., Moore S., Shi Y., Collins T. (1997). CREB-binding protein/p300 are transcriptional coactivators of p65. Proc. Natl. Acad. Sci. USA.

[76] Shishodia S., Amin H.M., Lai R., Aggarwal B.B. (2005). Curcumin (diferuloylmethane) inhibits constitutive NF-kappaB activation, induces G1/S arrest, suppresses proliferation, and induces apoptosis in mantle cell lymphoma. Biochem. Pharmacol..

[77] Yun J.M., Jialal I., Devaraj S. (2011). Epigenetic regulation of high glucose-induced proinflammatory cytokine production in monocytes by curcumin. J. Nutr. Biochem..

[78] El-Moselhy M.A., Taye A., Sharkawi S.S., El-Sisi S.F., Ahmed A.F. (2011). The antihyperglycemic effect of curcumin in high fat diet fed rats. Role of TNF-alpha and free fatty acids. Food Chem. Toxicol..

[79] Chiu J., Khan Z.A., Farhangkhoee H., Chakrabarti S. (2009). Curcumin prevents diabetes-associated abnormalities in the kidneys by inhibiting p300 and nuclear factor-kappaB. Nutrition.

[80] Chuengsamarn S., Rattanamongkolgul S., Luechapudiporn R., Phisalaphong C., Jirawatnotai S. (2012). Curcumin extract for prevention of type 2 diabetes. Diabetes Care.

[81] Kanwar J., Taskeen M., Mohammad I., Huo C., Chan T.H., Dou Q.P. (2012). Recent advances on tea polyphenols. Front. Biosci. (Elite Ed.).

[82] Lambert J.D., Yang C.S. (2003). Mechanisms of cancer prevention by tea constituents. J. Nutr..

[83] Thakur V.S., Gupta K., Gupta S. (2012). Green tea polyphenols causes cell cycle arrest and apoptosis in prostate cancer cells by suppressing class I histone deacetylases. Carcinogenesis.

[84] Choi K.C., Jung M.G., Lee Y.H., Yoon J.C., Kwon S.H., Kang H.B., Kim M.J., Cha J.H., Kim Y.J., Jun W.J. (2009). Epigallocatechin-3-gallate, a histone acetyltransferase inhibitor, inhibits EBV-induced B lymphocyte transformation via suppression of RelA acetylation. Cancer Res..

[85] Chen Y.K., Cheung C., Reuhl K.R., Liu A.B., Lee M.J., Lu Y.P., Yang C.S. (2011). Effects of green tea polyphenol (−)-epigallocatechin-3-gallate on newly developed high-fat/western-style diet-induced obesity and metabolic syndrome in mice. J. Agric. Food Chem..

[86] Chen L.F., Mu Y., Greene W.C. (2002). Acetylation of RelA at discrete sites regulates distinct nuclear functions of NF-kappaB. EMBO J..

[87] Lu C., Zhu W., Shen C.L., Gao W. (2012). Green tea polyphenols reduce body weight in rats by modulating obesity-related genes. PLoS One.

[88] Lambert J.D., Kennett M.J., Sang S., Reuhl K.R., Ju J., Yang C.S. (2010). Hepatotoxicity of high oral dose (−)-epigallocatechin-3-gallate in mice. Food Chem. Toxicol..

[89] Mazzanti G., Menniti-Ippolito F., Moro P.A., Cassetti F., Raschetti R., Santuccio C., Mastrangelo S. (2009). Hepatotoxicity from green tea: A review of the literature and two unpublished cases. Eur. J. Clin. Pharmacol..

[90] Arora T., Sharma R., Frost G. (2011). Propionate. Anti-obesity and satiety enhancing factor?. Appetite.

[91] Wolever T.M., Josse R.G., Leiter L.A., Chiasson J.L. (1997). Time of day and glucose tolerance status affect serum short-chain fatty acid concentrations in humans. Metabolism.

[92] Gao Z., Yin J., Zhang J., Ward R.E., Martin R.J., Lefevre M., Cefalu W.T., Ye J. (2009). Butyrate improves insulin sensitivity and increases energy expenditure in mice. Diabetes.

[93] Boffa L.C., Vidali G., Mann R.S., Allfrey V.G. (1978). Suppression of histone deacetylation *in vivo* and *in vitro* by sodium butyrate. J. Biol. Chem..

[94] Sengupta S., Muir J.G., Gibson P.R. (2006). Does butyrate protect from colorectal cancer?. J. Gastroenterol. Hepatol..

[95] Lin H.V., Frassetto A., Kowalik E.J., Nawrocki A.R., Lu M.M., Kosinski J.R., Hubert J.A., Szeto D., Yao X., Forrest G. (2012). Butyrate and propionate protect against diet-induced obesity and regulate gut hormones via free fatty acid receptor 3-independent mechanisms. PLoS One.

[96] Vinolo M.A., Rodrigues H.G., Festuccia W.T., Crisma A.R., Alves V.S., Martins A.R., Amaral C.L., Fiamoncini J., Hirabara S.M., Sato F.T. (2012). Tributyrin attenuates obesity-associated inflammation and insulin resistance in high-fat-fed mice. Am. J. Physiol. Endocrinol. Metab..

[97] Li H., Gao Z., Zhang J., Ye X., Xu A., Ye J., Jia W. (2012). Sodium butyrate stimulates expression of fibroblast growth factor 21 in liver by inhibition of histone deacetylase 3. Diabetes.

[98] Potthoff M.J., Inagaki T., Satapati S., Ding X., He T., Goetz R., Mohammadi M., Finck B.N., Mangelsdorf D.J., Kliewer S.A. (2009). FGF21 induces PGC-1alpha and regulates carbohydrate and fatty acid metabolism during the adaptive starvation response. Proc. Natl. Acad. Sci. USA.

[99] Yaku K., Enami Y., Kurajyo C., Matsui-Yuasa I., Konishi Y., Kojima-Yuasa A. (2012). The enhancement of phase 2 enzyme activities by sodium butyrate in normal intestinal epithelial cells is associated with Nrf2 and p53. Mol. Cell. Biochem..

[100] Kim S.C., Sprung R., Chen Y., Xu Y., Ball H., Pei J., Cheng T., Kho Y., Xiao H., Xiao L. (2006). Substrate and functional diversity of lysine acetylation revealed by a proteomics survey. Mol. Cell.

[101] Zhao S., Xu W., Jiang W., Yu W., Lin Y., Zhang T., Yao J., Zhou L., Zeng Y., Li H. (2010). Regulation of cellular metabolism by protein lysine acetylation. Science.

[102] Glozak M.A., Sengupta N., Zhang X., Seto E. (2005). Acetylation and deacetylation of non-histone proteins. Gene.

